# Immune responses in mice vaccinated with a DNA vaccine expressing serine protease-like protein from the new-born larval stage of *Trichinella spiralis*

**DOI:** 10.1017/S0031182016002493

**Published:** 2017-01-10

**Authors:** JING XU, XUE BAI, LI BO WANG, HAI NING SHI, JOKE W. B. VAN DER GIESSEN, PASCAL BOIREAU, MING YUAN LIU, XIAO LEI LIU

**Affiliations:** 1 Key Laboratory for Zoonoses Research, Ministry of Education, Institute of Zoonoses, Jilin University, Changchun, People's Republic of China; 2 Mucosal Immunology Laboratory, Pediatric Gastroenterology Unit, Massachusetts General Hospital East, Massachusetts, USA; 3 Centre for Infectious Disease Control, National Institute for Public Health and the Environment, Amsterdam, The Netherlands; 4 Laboratory for Animal Health, ANSES, INRA, ENVA, Universite Paris Est, Maisons Alfort, France; 5 Jiangsu Co-innovation Center for Prevention and Control of Important Animal Infectious Diseases and Zoonoses, Yangzhou, Jiangsu, People's Republic of China

**Keywords:** *Trichinella spiralis*, DNA vaccine, protective immunity, new-born larvae

## Abstract

*Trichinella spiralis* is a parasitic helminth that can infect almost all mammals, including humans. *Trichinella spiralis* infection elicits a typical type 2 immune responses, while suppresses type 1 immune responses, which is in favour of their parasitism. DNA vaccines have been shown to be capable of eliciting balanced CD4^+^ and CD8^+^ T cell responses as well as humoral immune responses in small-animal models, which will be advantage to induce protective immune response against helminth infection. In this study, serine protease (Ts-NBLsp) was encoded by a cDNA fragment of new-born *T. spiralis* larvae, and was inserted after CMV promoter to construct a DNA vaccine [pcDNA3·1(+)-Ts-NBLsp]. Ts-NBLsp expression was demonstrated by immunofluorescence. Sera samples were obtained from vaccinated mice, and they showed strong anti-Ts-NBLsp-specific IgG response. Mice immunized with the pcDNA3·1(+)-Ts-NBLsp DNA vaccine showed a 77·93% reduction in muscle larvae (ML) following challenge with *T. spiralis* ML. Our results demonstrate that the vaccination with pcDNA3·1(+)-Ts-NBLsp plasmid promoted the balance of type 1 and 2 immune responses and produced a significant protection against *T. spiralis* infection in mice.

## INTRODUCTION

*Trichinella* spp. are intestinal nematode parasites that can cause trichinellosis in humans and animals (Dupouy-Camet, 2000). More than 100 species of mammals, birds and reptiles can be infected by *T. spiralis* (Pozio and Zarlenga, 2013); domestic pigs, horses, dogs and cats are known as the most important hosts of *T. spiralis*. Infection occurs when humans consume raw or undercooked meat of different animal origins containing *T. spiralis* muscle larvae (ML). Trichinellosis is a public health hazard, and it also an economic problem in animal production and food safety (Dorny *et al.* 2009). It is difficult to control this zoonosis due to its wide distribution of domestic and wild animal reservoirs (Wang and Cui, 2001; Wang *et al.* 2006; Cui *et al.* 2011; Murrell and Pozio, 2011). So far, useful and stable anti-*Trichinella* vaccines that can be used in animal husbandry have not been developed yet. Therefore, it is necessary to develop a vaccine to prevent *Trichinella* infection in domestic animals and humans.

*Trichinella spiralis* is a nematode parasite that spends its larval and adult life stages in the same host. It has three major antigenic stages, ML, adult worms (AD) and new-born larvae (NBL). During natural course of *T. spiralis* infection, AD get pregnant and NBL are released in the intestines of the host, then all the AD evacuate from the host through intestines between 10 and 15 days post-infection (dpi). The NBL arrive at striated muscle through lymphatic vessels and blood circulation, and then develop into encapsulated ML in 20 days. Then ML start long-term parasitizing in striated muscle (Pozio, 1989; Gottstein *et al.* 2009). NBL is a key stage for the growth of *T. spiralis*, without the protection of capsule, larvae will be exposed to muscle immune system. Therefore, it is necessary to think highly of antigens from the NBL stage.

A highly antigenic NBL stage-specific serine protease gene, Ts-NBLsp, was obtained via a subtractive cDNA library of *T. spiralis* NBL (Liu *et al.* 2007). Ts-NBLsp showed encouraging potential in the early detection of *Trichinella* infection, but the function of it *in vivo* is unclear. Multiple serine proteases have been identified at different stages of *T. spiralis*, evidences have shown that serine proteinases are abundant in excretory/secretory products or crude extract proteins from *T. spiralis*. Most of these serine proteases are involved in parasite survival and establishment of infection (Todorova, 2000; Todorova and Stoyanov, 2000; Bien *et al.* 2012; Wang *et al.* 2014). Furthermore, serine proteases are involved in reproduction and evasion of the host immune system (Dzik, 2006; Balasubramanian *et al.* 2010). It has been reported that the antibody response against serine proteases from parasite can inhibit the protease activity and possibly contribute to inhibit invasion of the parasite in a sensitized host (Ros-Moreno *et al.* 2000; Todorova and Stoyanov, 2000).

Therefore, in this study the plasmid of pcDNA3·1(+)-Ts-NBLsp was constructed, purified and used as an experimental DNA vaccine to immunize Kunming mice, and to evaluate the immune response and protective effects in a murine model of *T. spiralis* infection.

## MATERIALS AND METHODS

### Parasites and animals

*Trichinella spiralis* (ISS534) used in this study was maintained in Wistar rats in our laboratory by serial passage infections. Muscle larvae were recovered from mice 35 dpi with artificial digestion solution (1% pepsin/HCl) (Li *et al.* 2010). Female Kunming mice aged 6–8 weeks were obtained from Norman Bethune University of Medical Science (NBUMS), China.

### Amplify the cDNA of Ts-NBLsp

The complete sequence of Ts-NBLsp gene has been submitted to Genebank (GenBank AY491941·1). The full-length cDNA of Ts-NBLsp was amplified by PCR from a plasmid of cDNA library of *T. spiralis* NBL with the following primers: 5-CCGGTACCGCGTTTGAATGCGGTGTGCC-3 (forward) and 5-GCGCTCGAGTTACTTAGAAAAGTGATA-3 (reverse). The *KpnI* and *XhoI* restriction sites are underlined. The PCR cycles consisted of an initial denaturation step at 94 °C for 5 min, 30 cycles of denaturation at 94 °C for 1 min and annealing at 53 °C for 1 min with an extension at 72 °C for 1 min, and a final extension at this same temperature for another 10 min.

### Recombinant protein rTs-NBLsp expression, purification and identified

The DNA fragment of Ts-NBLsp, described above, was cloned into pET28a expression vector using the T7 promoter. The recombinant plasmid pET28a/Ts-NBLsp was determined by DNA sequencing and transformed in *Escherichia coli* BL21 (DE3) chemically competent cells. The BL21 cells containing pET28a/Ts-NBLsp grown in 1 L LB and were shaken vigorously of 120 rpm at 37 °C up to an optical density of 0·6, calculated at 600 nm. Then, the cells were induced with isopro-pyl-b-D-thiogalactopyranoside (IPTG) to a final concentration of 1 mm with vigorous shaking of 120 rpm at 37 °C for 5 h. The cells were centrifuged at 7000 ***g*** for 15 min and the pellet was resuspended with binding buffer (20 mm Tris–HCl, pH: 7·9, 0·5 M NaCl, 8 M urea, 1 mm PMSF). Purification of the recombinant protein was performed as previously described (Feng *et al.* 2013). Briefly, the inclusion body was purified by Ni-affinity chromatography and refolded by drop-wise dilution. Then sodium dodecyl sulphate–polyacrylamide gel electrophoresis (SDS–PAGE) and Western blotting were used to identify the protein. Briefly, the protein was separated by 10% SDS–PAGE and electrophoretically transferred onto a nitrocellulose membrane (Bio-Rad, Hercules, CA, USA). The membrane was blocked with 5% skimmed milk in PBS containing 0·05% Tween-20 (PBST) overnight at 4 °C and incubated with mouse anti-Ts-NBLsp mAbs (1:500, this monoclonal antibody to Ts-NBLsp was produced by Laboratory for Animal Health, Maisons Alfort, France) for 1 h at 37 °C. HRP (horseradish peroxidase)-conjugated goat anti-mouse IgG (Beijing Dingguo Changsheng Biotechnology CO.LTD) was used as the secondary antibody. Finally, the protein bands were developed using ECL (enhanced chemiluminescence) reagents (Applygen Technologies Inc., Beijing, China). The images were photographed in a Chemi Doc image scanner from Bio Rad.

### Plasmid DNA vaccine constructs

The DNA fragment of Ts-NBLsp, described above, was cloned into the eukaryotic expression vector pcDNA3·1 (+) using the cytomegalovirus promoter. The sequence accuracy of recombinant plasmid was authenticated by double digestion and DNA sequencing. The recombinant plasmid was then purified from transformed *E. coli* DH5α cells by Endofree Plasmid Midiprep Kit (BioMIGA, San Diego, USA) following the manufacturer's instructions, dissolved in sterile endotoxin-free TE buffer and stored at −20 °C. The recombinant plasmid was named pcDNA3·1(+)-Ts-NBLsp.

### Immunization and challenge

Six-week-old female Kunming mice (20 per experimental group) were immunized (by bilateral intramuscular (IM) injection into the quadriceps) twice with 2 weeks interval. In pcDNA3·1(+)-Ts-NBLsp-vaccinated group, each mouse was injected with 60 *µ*g plasmid dissolved in 100 *µ*L sterile PBS at each immunization. As negative control, one group of mice were injected with 60 *µ*g empty pcDNA3·1(+)vector, and another group of mice injected with 100 *µ*L sterile PBS constituted the blank control. Two weeks after the last immunization, mice of each group were challenged with 250 *T. spiralis* ML. Blood was collected from the tail vein before and weekly after vaccination until the challenge infection, and sera samples were separated and stored at −20 °C. Pre-immune serum samples were used as negative controls.

### Detection of target gene expression in immunized mice by immunofluorescence test

To determine the expression of recombinant Ts-NBLsp *in vivo*, quadriceps femoris of three mice in each group were obtained at 48 h post the first immunization. These tissues were fixed in 4% paraformaldehyde (v/v) in PBS, embedded in paraffin, and cut into 3 *µ*m paraffin sections, and then immunofluorescence test (IFT) was performed as described previously. Briefly, the sections were incubated with 0·1% Triton X-100 in PBS at 4 °C for 1 h, blocked for non-specific protein binding by incubation in 10% goat serum diluted in PBS at room temperature for 1 h, and incubated with mouse anti-Ts-NBLsp mAbs (1:200 dilution) at 37 °C for 1 h. After washing, the sections were incubated with a 1:100 dilution of FITC-conjugated goat anti-mouse IgG (Santa Cruz, USA) at 37 °C for 1 h, and then stained with Hoechst (Beyotime Biotechnology, Beijing, China) for 5 min at room temperature. The sections incubated with serum from an unimmunized mouse at the same dilution served as a negative control. The sections were examined and photographed under fluorescent microscope (Olympus, Japan).

### Determination of antibodies

Anti-Ts-NBLsp antibodies (total IgG, IgG1 and IgG2a) in serum of immunized mice were checked by enzyme-linked immunosorbent assay (ELISA). Briefly, microtitre plates (Corning, Acton, MA) were coated with 100 *µ*L of the recombinant protein (rTs-NBLsp) (50 *µ*g mL^−1^ in coating buffer, overnight at 4 °C). The plates were washed with PBST and blocked with 5% non-fat dry milk (Sigma, St. Louis, MO) in PBST at 37 °C for 2 h, followed by incubation at 37 °C for 2 h with 100 *µ*L of the serum samples with a 1:50 dilution in PBST with 5% non-fat dry milk. After three washes, the plates were incubated at 37 °C for 2 h with 100 *µ*L well^−1^ of horseradish peroxidase-conjugated goat anti-mouse IgG antibody (Beijing Dingguo Changsheng Biotechnology Co.Ltd.) at a 1:4000 dilution, or goat anti-mouse IgG1 or goat anti-mouse IgG2a (Abcam, Cambridge, UK) at a 1:500 dilution, which was used for determination of total IgG antibody levels and isotype analysis, respectively. ELISA was developed by chromogen 3,3′,5′,5′-tetramethylbenzidine (TMB, Tiangen Biotech Co., Beijing, China), the reaction was terminated by 2 N H_2_SO4 solution at 15 min after substrate addition, and the OD 450 (Optical density at 450 nm) value was measured.

### Cytokine assays

To analysis the cellular immune responses of mice immunized with DNA construct, the concentrations of interleukin 4 (IL-4), IL-10 and interferon (IFN)-*γ* in serum sample collected before and weekly after vaccination were measured by ELISA according to the manufacturer's instructions (eBioscience, San Diego, California). The concentration of cytokines was determined by comparison with the standard curves constructed with known amounts of the respective mouse recombinant cytokines. Results were expressed in picograms per millilitre (pg mL^−1^).

### Flow cytometry analysis of T lymphocytes

One week after the final immunization, blood was collected from five mice in each group and poured slowly into anticoagulation tubes. Staining of the cells for flow cytometry analysis was performed by direct staining. Briefly, the cells were stained with optimal concentrations of APC-conjugated Hamster anti-mouse CD3e (0·2 mg mL^−1^, clone 145–2C11, BD Biosciences, San Jose, CA, USA), FITC-conjugated Rat anti-mouse CD4 (0·5 mg mL^−1^, clone GK 1·5, BD Biosciences, San Jose, CA, USA), and PE-conjugated Rat anti-mouse CD8a (0·2 mg mL^−1^, clone 53–6·7, BD Biosciences, San Jose, CA, USA), and incubated at room temperature for 30 min. Red blood cells were lysed by the addition of a triple volume of lysis solution (Solarbio, Beijing, China) on ice for 15 min. Cells were then washed twice in PBS, suspended in 300 *µ*L PBS, and immediately analysed with a BD FACSCalibur™ flow cytometer (BD Biosciences, Heidelberg, Germany).

### Evaluation of immune protection

Twelve mice of each group were sacrificed 42 days after challenge, the carcass weight and the ML of each mouse were examined. The protective immunity was calculated as the worm reduction rate of recovered ML per gram from the vaccinated group compared with the blank control group.

### Statistical analysis

All statistical analyses were performed by SPSS 16·0 Data Editor. Data were expressed as the mean ± standard deviation (s.d.), and the differences of the data between all the groups were evaluated by one-way ANOVA analysis. The difference between groups were regarded as statistically significant if *P* < 0·05.

## RESULTS

### *Identification plasmids of pcDNA3·1*(*+*)*-Ts-NBLsp and pET28a/Ts-NBLsp*

The constructed plasmids were subjected to digestion with *KpnI* and *XhoI*. Electrophoretic separation of the digestion products showed that the construction of the recombinant plasmids was successful (data not shown). DNA sequence analysis indicated that the amplified fragment of Ts-NBLsp gene consisting of 1209 bp was correctly cloned into prokaryotic expression vector pet28a and eukaryotic expression vector pcDNA3·1(+).

### Recombinant protein rTs-NBLsp was identified by Western blot

**Fig. 1.**
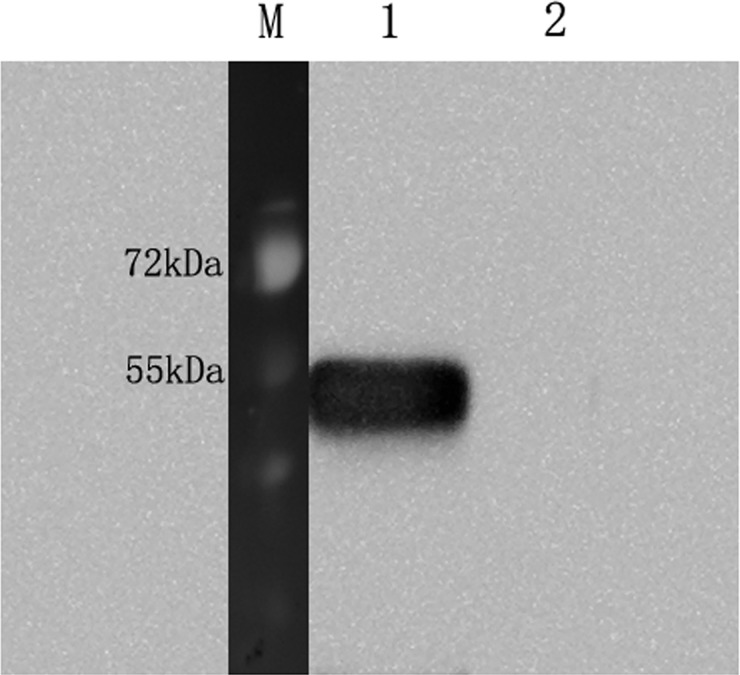
Analysis of the Recombinant protein rTs-NBLsp by Western blot. The rTs-NBLsp fusion protein purified from pET28a/Ts-NBLsp plasmid transformed *E. coli* BL21 was recognized by mouse anti-Ts-NBLsp mAbs (lane 1), but fusion protein purified from control group [pET28a (+) plasmid transformed *E. coli* BL21] was not recognized by mouse anti-Ts-NBLsp mAbs (lanes 2).

Western blot results demonstrated that the expression of recombinant protein rTs-NBLsp could be induced after addition of IPTG. The mouse anti-Ts-NBLsp monoclonal antibody recognized the recombinant protein, which is around 44·46 kDa in recombinant plasmid pET28a/Ts-NBLsp transformed *E. coli* cells, no protein band was observed in the pET28a transformed *E. coli* cells. (Fig. 1).

### Ts-NBLsp was identified in vaccinated mice by immunofluorescence

**Fig. 2.**
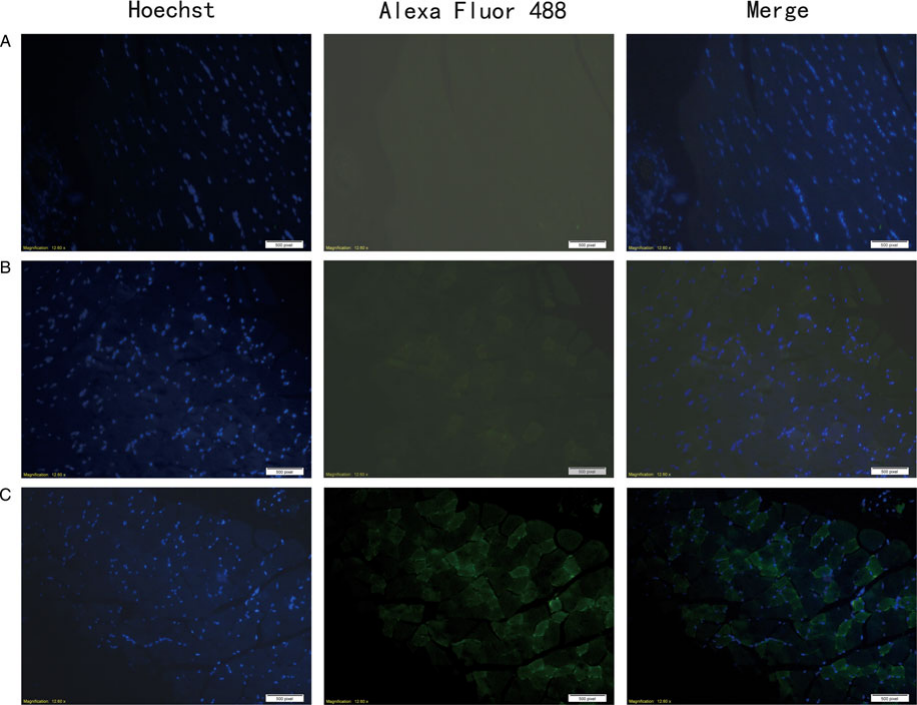
Detection of pcDNA3·1(+)-Ts-NBLsp expression in mouse skeletal muscles by an immunofluorescence test. Tissue sections from (A) PBS control group; (B) pcDNA3·1(+)-immunized mice; (C) pcDNA3·1(+)-Ts-NBLsp-immunized mice at 48 h post first immunization were detected by mouse anti-Ts-NBLsp mAbs.

In immunofluorescence analysis, after incubated with mouse anti-Ts-NBLsp mAbs, the sections from mouse vaccinated with plasmid of pcDNA3·1(+)-Ts-NBLsp at 48 h showed specific green fluorescence. However, the negative control, which were vaccinated with pcDNA3·1(+) or PBS, did not show any fluorescence emission (Fig. 2). These results demonstrate that the Ts-NBLsp protein was expressed in quadriceps femoris of the mice vaccinated with pcDNA3·1(+)-Ts-NBLsp, where it retained its antigenic reactivity.

### Humoral immune responses induced by vaccination

**Fig. 3.**
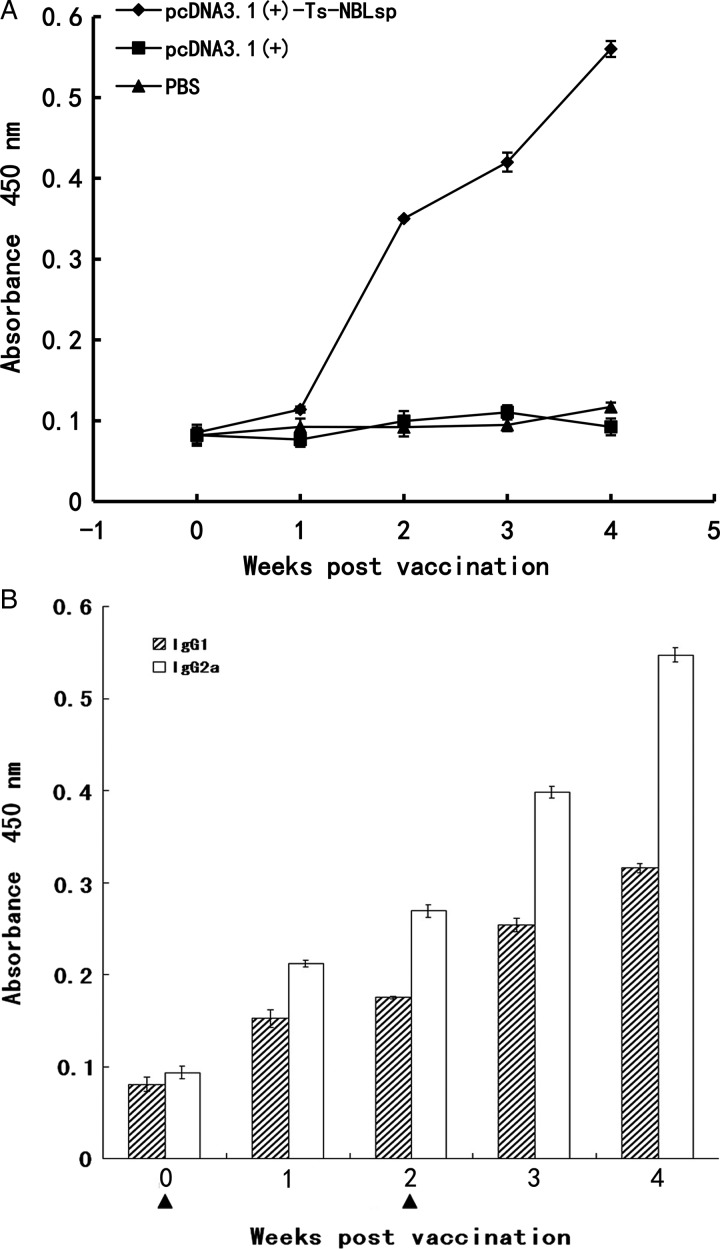
Detection of serum IgG and IgG isotypes. Mouse IgG (A) and IgG subclass (B) responses to the Ts-NBLsp were measured by ELISA. Values shown for each group are the mean ± s.d. of antibody levels (*n* = 12). The immunization time points are marked as solid triangle (▲).

As shown in (Fig. 3), specific anti-Ts-NBLsp IgG has been induced in pcDNA3·1(+)-Ts-NBLsp-vaccinated group. The analysis of the IgG isotypes showed that in the pcDNA3·1(+)-Ts-NBLsp-vaccinated group, the IgG2a-predominated IgG antibody elevation suggesting a Th1-predominated Th1/Th2 mixed immune response had been induced.

### Evaluation of the phenotypic change of the T lymphocytes

**Fig. 4.**
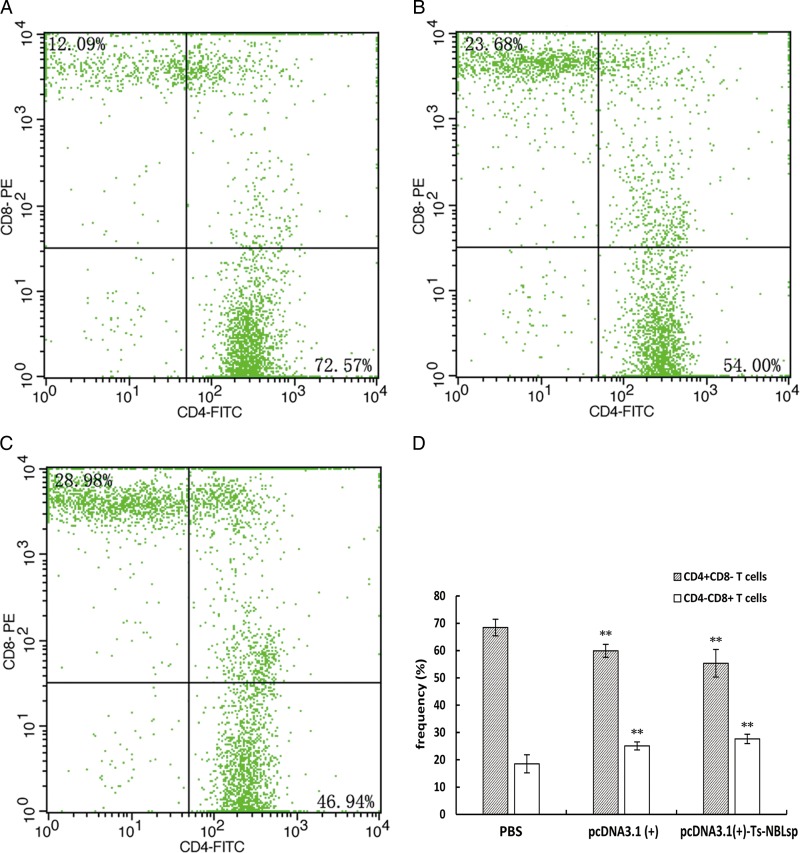
The detection of peripheral blood T lymphocytes by flow cytometry. The percent change of CD4^+^and CD8^+^T cells in peripheral blood lymphocytes from (A) PBS-control mice, (B) pcDNA3·1 (+)-immunized mice and (C) pcDNA3·1(+)-Ts-NBLsp-immunized mice. The upper left and lower right quadrants of each panels show the CD3^+^/CD8^+^ and CD3^+^/CD4^+^ double positive cells, respectively. (D) The data summary of three groups, with data presented as the mean ± s.d., *n* = 5. Asterisks (**) indicate statistically extremely significant differences between pcDNA3·1(+)-Ts-NBLsp and PBS or between pcDNA3·1(+) and PBS (*P* < 0·01).

One week after the final immunization, when compared with the control group [PBS and pcDNA3·1(+)], the CD4^+^ T lymphocyte count was significantly reduced (*P* < 0·01), while the CD8^+^ T lymphocyte count was significantly increased (*P* < 0·01). The CD4^+^/CD8^+^ ratio (55·36:27·66 = 2·00) was decreased in the pcDNA3·1(+)-Ts-NBLsp-vaccinated group compared with PBS-vaccinated group (68·45:19·41 = 3·53) (Fig. 4).

### Evaluation of cytokine production

**Fig. 5.**
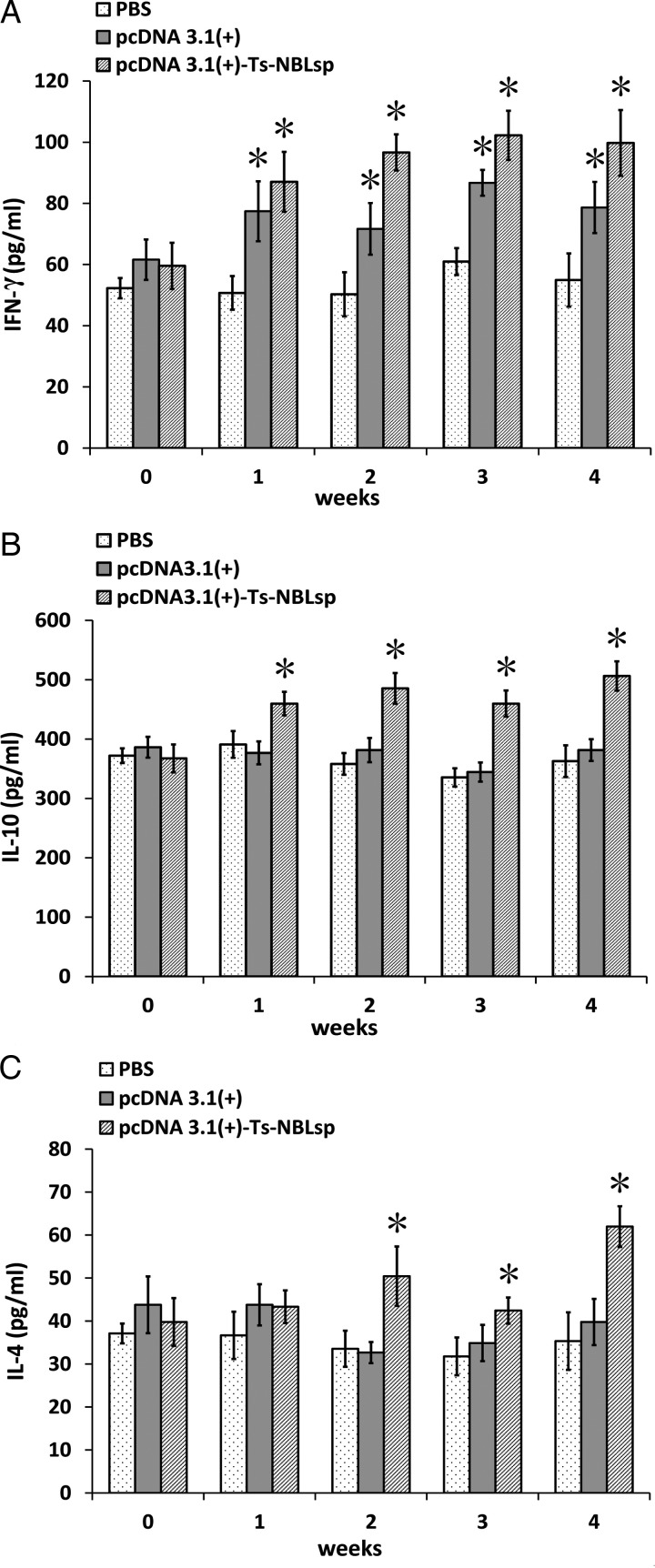
Detection of the production of cytokines with ELISA. IFN-*γ* (A), IL-10 (B) and IL-4 (C) upon Ts-NBLsp stimulation were detected by ELISA. Data are presented as the mean ± s.d. of 12 mice per group. Asterisks (*) indicate statistically significant differences between pcDNA3·1(+)-Ts-NBLsp and PBS or between pcDNA3·1(+) and PBS (*P* < 0·05).

As shown in Fig. 5, compared with the control group (PBS), in the pcDNA3·1(+)-Ts-NBLsp-vaccinated group and pcDNA3·1(+)-vaccinated group, the levels of cytokine of Th1-type (IFN-*γ*) were all significantly increased (*P* *<* 0·05); however, the levels of Th2-type cytokines (IL-10 and IL-4) were significantly increased (*P* *<* 0·05) only in pcDNA3·1(+)-Ts-NBLsp-vaccinated group.

### Assessment of protective effects of DNA vaccine

**Fig. 6.**
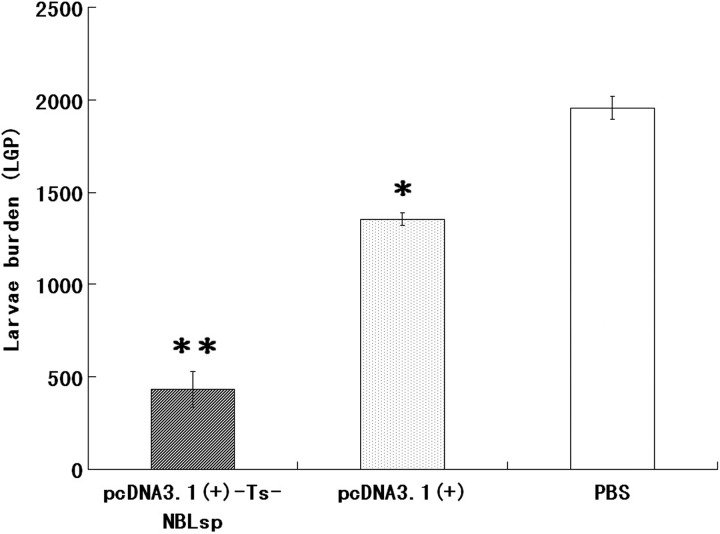
Protective immunity of pcDNA3·1(+)-Ts-NBLsp-vaccinated mice after being challenged with 250 *Trichinella spiralis* larvae. The results are presented as the arithmetic mean of 12 mice per group ± s.d. (*P < 0·05, ** *P* < 0·01).

The pcDNA3·1(+)-Ts-NBLsp-immunized group exhibited a 77·93% reduction in the ML burden 42 days after challenge relative to the blank control group (PBS) (Fig. 6). Compared with the PBS-vaccinated control mice, the pcDNA3·1(+)-immunized group exhibited 30·34% reduction in ML burden.

## DISCUSSION

DNA vaccines can express antigens in proper conformation with post-translational modifications and intracellular trafficking (Ulmer *et al.* 1993; Xiang *et al.* 1994; Boudinot *et al.* 1998). In the present study, we constructed the plasmid of pcDNA3·1(+)-Ts-NBLsp and immunized mice by IM needle injection to mimic a NBL stage-specific serine protease expressed during natural infection process. The expression of recombinant Ts-NBLsp *in vivo* was confirmed by immunofluorescence test, the recombinant Ts-NBLsp was distributed on the inner surface of the membrane of the muscle cells in quadriceps femoris of pcDNA3·1(+)-Ts-NBLsp-vaccinated mice at 48 h post first immunization.

The phenotypic change of T lymphocytes in peripheral blood was evaluated one week after the final immunization, and compared to the control group (PBS). The CD8^+^ T lymphocyte count significantly increased (*P* *<* 0·01) in both pcDNA3·1(+)-Ts-NBLsp-vaccinated mice and pcDNA3·1(+)-vaccinated mice, and no significant difference was observed between this two groups. Furthermore, the IFN-*γ* levels in serum were significantly increased (*P* *<* 0·05) in both groups. It indicated that in the present study the DNA vaccine has triggered the non-specific immune response. In fact, it has been shown that immune response induced by DNA vaccine is not dependent solely on the expression of antigen, and the DNA molecule itself can act as an adjuvant to enhance the immune response in mammals (Klinman *et al.* 1997; Krieg *et al.* 1998). The result of the present study shows that the pcDNA3·1(+)-vaccinated group exhibited a 30·34% reduction in the ML burden 42 days after challenge compared to the control group. It is suggesting that non-specific immune responses might be useful for the protection against the ML of *T. spiralis*.

There are two major effector arms of the adaptive immune system, antibodies and T cells, and both are important for resistance to primary infections. The pcDNA3·1(+)-Ts-NBLsp-vaccinated group exhibited a 77·93% reduction in the ML burden. The antibodies and T cells response have been analysed. Compared to the control groups, serum IgG were significantly increased after boost immune in this group, and the IgG isotypes, both IgG1 and IgG2a, were increased, with IgG2a taking the dominant place. Because the IgG isotypes are controlled by cytokines secreted by the CD4^+^T cells, the IgG2 isotype is considered to be associated with a Th1 immune response, whereas the IgG1 isotype is associated with a Th2 response. It is suggested that Th1 predominated Th1/Th2 mixed immune response has been induced in the pcDNA3·1(+)-Ts-NBLsp-vaccinated group.

For the cellular immunologic response, IL-4 is one of the key cytokines in induction of Th2 responses, and IL-10 is an important effector of Th2 responses. The increased expression of these two cytokines suggests that the specific CD4^+^ Th2 response has been induced. Furthermore, the expression of IFN-*γ* was significantly increased (*P* *<* 0·05) in the pcDNA3·1(+)-Ts-NBLsp-vaccinated group. IFN-*γ* is a key effector of CD4^+^ Type 1 and CD8^+^ T cell, it can activate macrophages and dendritic cells, stimulate increased expression of MHC–peptide complexes, and defend against the intracellular infection. *Trichinella* sp. are intracellular parasites of muscle cells, and the NBL are without the protection of a capsule. It has been reported that IFN-*γ* is crucially involved in protection against NBL (Helmby and Grencis, 2003).

*Trichinella spiralis* can evoke a stage specific protective host immune response through their cuticular and ES antigens of each stage (Wang, 1997). During *T. spiralis* intestinal infection, CD4^+^ Th2 cells are critical in host protective immune and inflammatory responses (Ha *et al.* 1983; Khan *et al.* 2001). However, at muscular phase of *T. spiralis* infection, Th1 mediated the destructive granulomatous response, while Th2 are largely not destructive to ML (Li and Ko, 2001; Beiting *et al.* 2007). Furthermore, Th2-type response will inhibit the Th1-type response. So, the pcDNA3·1(+)-Ts-NBLsp plasmid may have promoted Th1 and Th2 immune balance by inducing Th1 predominated Th1/Th2 mixed immune response.

In conclusion, Ts-NBLsp gene induced an effectively specific immune response in vaccinated mice. We presume that Ts-NBLsp may be an important gene for the survival of *T. spiralis*; further studies are required to elucidate the roles of this gene. Additionally, for the research on vaccine against trichinellosis, the DNA vaccine successfully promoted the balance of Th1 and Th2 response, and induced the activity of CD8^+^ T cells. We presume that these two factors may play an important role in producing a significant protection against *T. spiralis* infection in mice.

## CONFLICT OF INTEREST STATEMENT

None of the authors has any financial or personal relationship with other people or organizations that could inappropriately influence or bias the paper entitled ‘Immune responses in mice vaccinated with a DNA vaccine expressing serine protease-like protein from the NBL stage of *Trichinella spiralis*’.
